# A randomized trial involving a multifunctional diet reveals systematic lipid remodeling and improvements in cardiometabolic risk factors in middle aged to aged adults

**DOI:** 10.3389/fnut.2023.1236153

**Published:** 2023-09-14

**Authors:** Claudia Balderas Arroyo, Maider Greño Ocariz, Oksana Rogova, Mahmoud Al-Majdoub, Inger Björck, Juscelino Tovar, Peter Spégel

**Affiliations:** ^1^Centre for Analysis and Synthesis, Department of Chemistry, Lund University, Lund, Sweden; ^2^Unit of Molecular Metabolism, Department of Clinical Sciences in Malmö, Lund University, Malmö, Sweden; ^3^Retired, Lund, Sweden; ^4^Department of Food Technology, Engineering and Nutrition, Food for Health Science Centre Lund University, Lund, Sweden

**Keywords:** functional foods, metabolic syndrome, diabetes, cardiovascular disease, degree of unsaturation, carbon number

## Abstract

**Background:**

A multifunctional diet (MFD) combining foods and ingredients with proven functional properties, such as fatty fish and fiber-rich foods, among others, was developed and shown to markedly reduce cardiometabolic risk-associated factors.

**Objective:**

Here, we aim at examining metabolic physiological changes associated with these improvements.

**Methods:**

Adult overweight individuals without other risk factors were enrolled in an 8-week randomized controlled intervention following a parallel design, with one group (*n* = 23) following MFD and one group (*n* = 24) adhering to a control diet (CD) that followed the caloric formula (E%) advised by the Nordic Nutritional Recommendations. Plasma metabolites and lipids were profiled by gas chromatography and ultrahigh performance liquid chromatography/mass spectrometry.

**Results:**

Weight loss was similar between groups. The MFD and CD resulted in altered levels of 137 and 78 metabolites, respectively. Out of these, 83 were uniquely altered by the MFD and only 24 by the CD. The MFD-elicited alterations in lipid levels depended on carbon number and degree of unsaturation.

**Conclusion:**

An MFD elicits weight loss-independent systematic lipid remodeling, promoting increased circulating levels of long and highly unsaturated lipids.

**Clinical trial registration:**

https://clinicaltrials.gov/ct2/show/NCT02148653?term=NCT02148653&draw=2&rank=1, NCT02148653.

## 1. Introduction

The prevalence of cardiometabolic diseases (CMDs) is escalating (1). This imposes a huge burden on the health care system and results in substantial suffering for the affected individual. Hence, effective preventive strategies are urgently needed to ensure a more proactive health care system.

Epidemiological studies have highlighted unhealthy dietary patterns as a major risk factor for CMD (2). *In vitro* studies have identified and characterized a multitude of bioactive or functional foods and ingredients that may underlie beneficial effects on CMD risk (2). Some of these perceived effects have been confirmed in randomized controlled trials, although the potential disease-preventing power of individual foods generally is relatively small (3). An alternative is to combine several different food ingredients, each having a small but proven health effect, into a multifunctional diet (MFD) (4–7). MFD includes foods and meals with high contents of polyphenols and specific sources of dietary fiber (5). These ingredients reduce postprandial glucose and are rich in omega 3 fatty acids (e.g., oily fish and rapeseed oil), which lower the risk of cardiovascular disease. The MFD also contain bioactive foods exhibiting anti-oxidative, anti-inflammatory and anti-hypercholesterolaemic power (e.g., soybeans and almonds). The effect of a MFD on multiple health-related variables has been studied in overweight subjects without any other risk factor, revealing rapid, within 4 weeks, beneficial effects on blood lipids, with marked reductions in LDL cholesterol, total cholesterol and triglycerides, as well as minor reductions in other cardiometabolic risk-associated biomarkers (5, 6). The diet was also found to partially remodulate gut microbiota (4).

Hence, alterations in clinically assessed health-related variables suggest multiple beneficial health effects of MFD. However, these measurements provide a quite simplistic view of the health effects mediated by the MFD and a limited understanding of the underlying mechanisms. A deeper understanding can be obtained by measuring additional variables related to metabolism. In a previous 4-weeks intervention study, untargeted metabolomics was used to generate a more detailed picture of the metabolic effects of an MFD under conditions enforcing weight maintenance (7). The study revealed that the above-mentioned beneficial changes in CMD risk-related biomarkers were mainly associated with alterations in the lipid portion of the blood metabolome (7).

In a more recent study, the metabolic impact of MFD was evaluated in a cohort of overweight volunteers without additional risk factors during a longer intervention (8 week) following a randomized parallel design (6). In that study, the clinical effects elicited by the MFD were compared with those induced by a nutritionally adequate control diet devoid of the functional components of MFD (6). Importantly, the study was performed under conditions that allowed for changes in body weight, a central component of CMD risk, and confirmed the beneficial action of MFD on blood lipid profiles and blood pressure. The objective of this study was to assess alterations in the plasma lipidome and metabolome with a particular focus on defining systematic MFD-elicited metabolic remodeling.

## 2. Materials and methods

### 2.1. Participants

Characteristics of the study participants have been previously described in detail (6). The MFD group included 23 individuals (3 men, 20 females) and the CD group 24 individuals (9 men, 15 females). Briefly, the study included adult healthy volunteers, i.e., without any known medical condition, who were non-smoking, normoglycemic (fasting plasma glucose ≤6.1 mmoL/L), with age between 50 and 73 years, and overweight (body mass index in the 25–33 kg/m^2^ range). Non-variable hormone replacement therapy for thyroid diseases was accepted (two female subjects), as was eventual consumption of prescription-free pain-killers but not those with anti-inflammatory action. A consort flow diagram can be found in Supplementary Figure S1.

### 2.2. Study protocol and diets

The study protocol and the diets have been previously described in detail (5, 6). The characteristics of the diets are summarized in Table 1 [for a full description see reference (5)]. Briefly, the study compared MFD with a control diet (CD) that contained none or rather limited amounts of the functional ingredients present in MFD. A randomized, controlled, parallel design over 8 weeks was followed, where the diets were prescribed through detailed daily menus comprising the 3 main meals and snacks. The participants received the required amounts of the following food items: blueberries, cinnamon, phytostanol-containing margarine, soybeans, soy protein-based foods, canned fish, rapeseed oil, barley kernels, special guar gum-rich and whole grain-based barley breads, fiber-rich oat beverage, betaglucan-rich oat fiber cereal, and whey protein. Consumption of dietary supplements was stopped 2 weeks prior to initiation of this trial. The diets (CD/MFD) supplied 2130/2195 and 2690/2740 Kcal per day for women and men, respectively. The CD followed the caloric formula (E%) advised by the Nordic Nutritional Recommendations. Consumption of alcohol was limited during the test period, with an accepted consumption of 30 and 37 g/week for women and men, respectively. Supplementary Table S1 summarizes the amount of main food categories included in the two diets. Participants were examined and a fasted plasma sample collected at baseline, 4 and 8 weeks. The study was approved by the Regional Ethical Review Board, Lund, Sweden (Dnr 2013/584). The trial was registered at www.clinicaltrials.gov as NCT02148653.

**Table 1:** Nutritional profiles of CD and MFD and main functional action and average content of active components in MFD.

	CD		MFD	
	Women	Men	Women	Men
Energy (kcal/day)*	2,045	2,570	2,100	2,615
Protein (E%)	15	14	19	18
Carbohydrate (E%)	56	55	51	50
Fat (E%)	29	30	31	31
Saturated fat (E%)	12.8	13.2	5.9	5.9
Monounsaturated fat (E%)	10.5	11.1	13.0	13.6
Polyunsaturated fat (E%)	3.6	3.7	8.2	8.4
w-6 fatty acids (E%)	2.9	3.1	4.2	4.3
w-3 fatty acids (E%)	0.8	0.8	2.2	2.3
w-6/w-3 ratio	3.8	3.8	1.9	1.9
Dietary fiber (g/day)	22	26	49	61
Cholesterol (mg/day)	200	240	140	160

Food component	Main functional role	Content in MFD	
		(g/day)	
		Women	Men
Soybean/soy protein	Cholesterol-lowering, anti-inflammatory	21	25
Viscous fibers	Cholesterol- lowering, prebiotic, GI-reducing		
b-glucans		5.8	6.2
Guar gum		5.6	6.7
Total		11.4	12.9
Long chain w-3 fatty acids (20:5 + 22:6)	Triglyceride-lowering, anti-inflammatory	2.4	3.0
Almonds	Cholesterol-lowering	28	28
Plant stanols	Cholesterol-lowering	2.0	2.7
Cinnamon	Antioxidant	3.0	3.0
Blueberries	Antioxidant, prebiotic	74.5	94.5
Vinegar	GI -reducing	22.5	22.5
Whey protein**	GI-reducing	4.3	4.3

For further details see (6). ^*^Mean daily values. **Whey protein (10 g) was only consumed simultaneously with high glycaemic index. Meals (potatoes, parsnip), 3 times per week.

### 2.3. Routine blood tests

Routine blood tests were analyzed at Clinical Chemistry, Skåne University Hospital, Malmö and included total cholesterol, HDL, triglycerides ApoA1, ApoB, C-reactive protein (CRP), gamma-glutamyl transferase (GT), insulin and HbA1c. LDL concentrations were estimated, HOMA-IR calculated, and fasting capillary glucose, breath hydrogen, PAI-1, GLP-1 and GLP-1 measured as described previously (6).

### 2.4. Lipid and metabolite profiling

Lipids were extracted from 40 μL blood plasma using a two-phase system based on methyl tertbutyl ether (MTBE), methanol and water and redissolved in 50 μL of isopropanol/acetonitrile (90/10; v/v) immediately prior to analysis (8). Analyses was conducted using two methods, both employing an Acquity UPLC-CSH C18 column (1.7 μm, 2.1*100 mm; Waters Corporation, Milford MA), as previously described in detail (8). Samples were analyzed in a single batch using constrained randomization (9), with samples from the same individual analyzed as a block, with randomization both within and between blocks. Thereby, the impact of instrumental drift on the ability to detect effects of the diet on metabolite and lipid levels was minimized. Quality control (QC) samples, prepared by mixing aliquots from all lipid extracts, were analyzed 8 times prior to the first injection and then every tenth injection to monitor the stability of the system. Lipids were identified by MS/MS and retention time correlation using the MassHunter METLIN Metabolite PCDL (Agilent Technologies, Santa Clara, CA) and our in-house developed lipid database. Chromatograms were then aligned, mass spectra deconvoluted and peaks integrated using a targeted approach in MassHunter Profinder B.06.00 Build 6.0625.0 (Agilent) and the open-source software mzMine 2.2.3.

Metabolites were extracted from 40 μL blood plasma using a one-phase methanol-based protein precipitation protocol (10). Following methoxymation and trimethylsilylation, metabolites were analyzed on an Agilent 7,890 gas chromatograph connected to a 5,975 single quadrupole mass analyzer according to a previously published method (10). Data were exported as NetCDF files, chromatograms aligned and spectra deconvoluted using a hierarchical multivariate curve resolution (HMCR)-based script (11). Metabolites were identified based on retention index, derived from injection of a homologous series of n-alkanes, and mass spectral matching using the NIST MS library and in house libraries.

### 2.5. Statistical analysis

Statistical analyses were conducted in R version 3.6.1. Non-normally distributed data, as judged from the Shapiro–Wilk test of normality (shapiro.test, stats) were log2-transformed. Lipid and metabolite data were analyzed separately due to differences in variance structure. Principal component analysis (PCA) was conducted using prcomp (stats) on mean centered and unit variance scaled data. Differences between groups were asses using linear mixed-effect models (LMMs; lmer, lme4), using the individual as blocking factor and including the interaction between study occasion and diet type, evaluated using Anova (car) with a paired t-test (t.test, stats) *post hoc*. Frequency data were examined using the chi-squared statistics (chisq.test, stats). Correlations between changes in lipid and metabolite data and alterations in anthropometric and clinical data were assessed using complete pairwise Spearman correlations (cor and cor.test, stats). Linear models were calculated using lm (stats). Orthogonal projections to latent structures-discriminant analysis (OPLS-DA) were performed on mean centerd and unit variance scaled data using opls (ropls) with 9-fold cross-validation. Summary statistics was generated using describeBy (psych). Results were illustrated using ggplot2, factoextra, pheatmap, ggVennDiagram and sjPlot packages. Significance was defined as *q* > 0.05 using multiple testing adjustments according to the false discover rate method (p.adjust, stats).

## 3. Results

### 3.1. Impact of MFD on cardiometabolic risk markers

The effects of the 8-weeks intervention with MFD on clinical cardiometabolic risk-associated variables were reported elsewhere (6). In summary, weight loss was significantly associated with time (*p* < 2e-16) and did not differ between diets (*p* > 0.05 for both diet and the interaction between diet and time). Hence, both groups had lost on average 2 and 4 kg of body weight at the 4- and 8-weeks study visit, respectively. MFD improved a range of cardiometabolic risk markers while the CD did not have significant metabolic effects. Besides minor reductions in diastolic blood pressure, the intake of MFD resulted in remarkable beneficial changes in commonly assessed components of the blood lipid profile, namely LDL cholesterol (−34%), total cholesterol (−26%), LDL-to-HDL and Apo B-to-Apo A1 ratios (−27% and − 15%, respectively) and triglyceride (−16%) (6). These effects were independent of changes in body weight.

### 3.2. Diet elicited alterations in metabolite and lipid profiles

**Figure 1:**
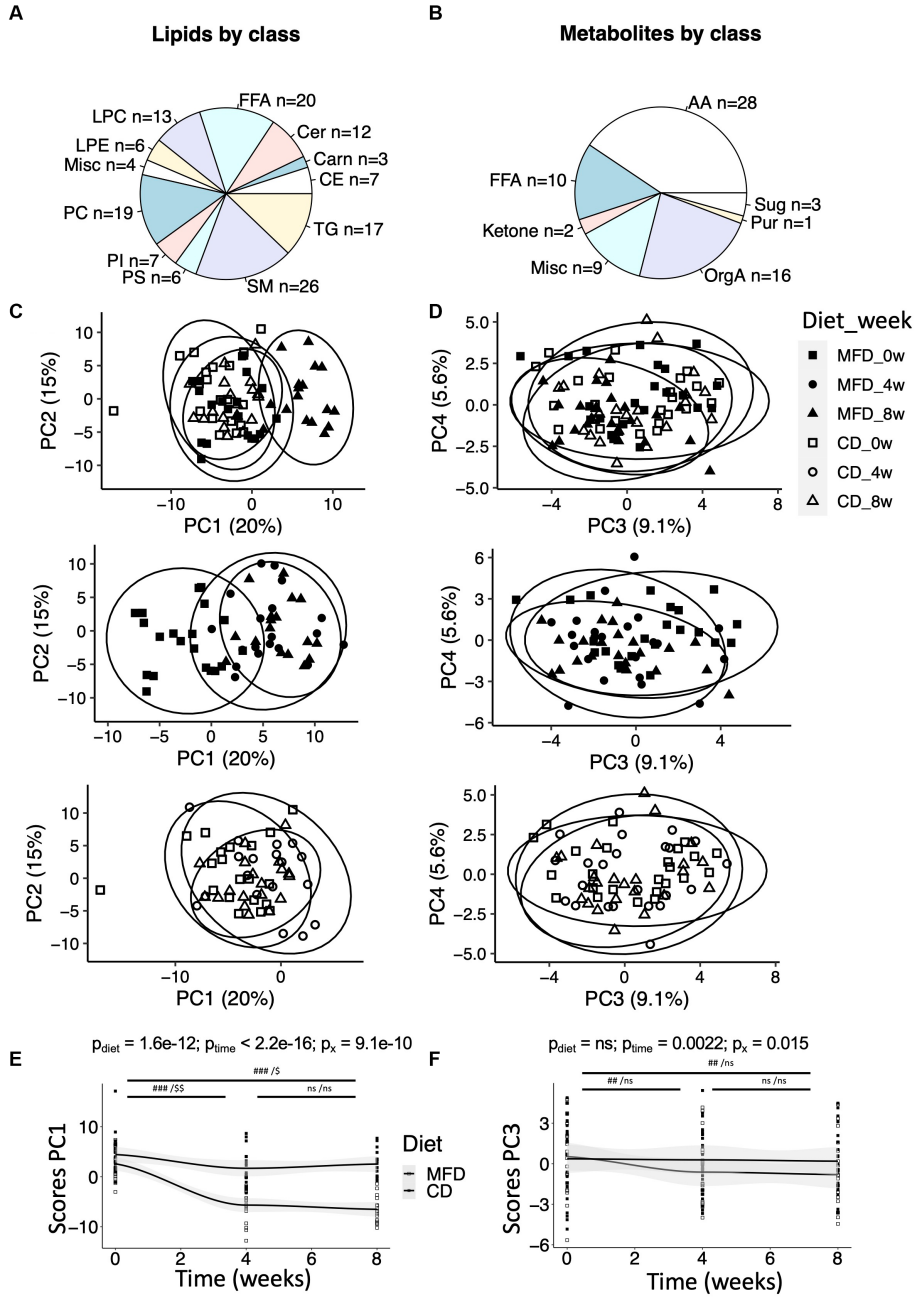
Overview of metabolites and their regulation during the two dietary regimens. **(A)** Distribution of lipids within lipid classes detected by the lipidomic method. Lipid classes are named by their standard abbreviations. The miscellaneous (Misc) group contains two bile acids and two furanpropionates. See Supplementary Table S2. **(B)** Distribution of metabolites detected by GC/MS within amino acids (AA), sugars (Sug), purines (Pur), organic acids (OrgA) and ketones. The Misc group contain metabolites such as cholesterol and various glycerol-related metabolites. See Supplementary Table S3. **(C)** Score plot from a principal component analysis (PCA) performed on the lipid data, and **(D)** score plot from a PCA performed on the metabolomic data. For clarity, the score plots have been broken down to show (top) weeks 0 and 8 for the MFD and CD, (center) MFD weeks 0, 4, and 8, and (bottom) CD weeks 0, 4, and 8. **(E)** Changes in scores along principal component 1 (PC1) over time for the lipidomic data, and **(F)** changes in scores along PC3 over time for the metabolomic data. Differences between time-points **(E,F)** were assessed by linear mixed effect models with the paired Student’s t-test *post hoc*: ###, *p* < 0.001 and ##, *p* < 0.01, for the multifunctional diet (MFD), and $$, *p* < 0.01 and $, *p* < 0.05, for the control diet (CD).

We detected 140 lipids and 69 low molecular weight metabolites using our three methods (Figures 1A,B). Data from the two methods applied to the lipid extracts were pooled. First, we produced an overview of alterations in the lipid and metabolite profiles using PCA. The lipid data revealed a clear time-dependent shift along principal component 1 (PC1) for the MFD group, whereas for the CD group, samples acquired at follow up remain clustered with the baseline samples (Figure 1C). A similar trend, although less distinct, could also be observed in PC3 for the low molecular weight metabolites measured by GC/MS (Figure 1D). Next LMMs were calculated for PC1 and 3 for the lipid and metabolite data, respectively, revealing significant diet-dependent score trajectories (interaction time*diet), for both lipid (*p* = 9.1e-10, Figure 1E) and metabolite data (*p* = 0.015, Figure 1F). The associations remained after adjustment for weight loss. The lipidome changed already at 4-weeks after initiation of the MFD and the CD and then did not change further between the 4- and 8-weeks study visits (Figure 1E). A similar result was obtained for the MFD with respect to the low molecular weight metabolites, but no changes were observed for the CD (Figure 1F).

### 3.3. Shared and unique alterations in the metabolite and lipid profiles elicited by the diets

Next, we assessed alterations in metabolite and lipid levels elicited by the two diets using LMMs calculated individually for each metabolite and lipid. The models included an interaction term, allowing us to identify metabolites and lipids that are altered differently depending on the diet. This analysis revealed 62 lipids and 7 low molecular weight metabolites to show significant interactions between diet and time (Supplementary Tables S2, S3; Supplementary Figures S2, S3, for lipidomics and metabolomics data, respectively). Notably, all of the 12 detected ceramides and 12 out of the 19 measured PCs showed significant interactions. Among metabolites detected by GC/MS, four out of the seven metabolites showing significant interactions, i.e., cholesterol and heptadecanoic-, stearic- and lauric acid, were related to lipid metabolism. All interactions remained significant after adjustment for weight loss.

**Figure 2:**
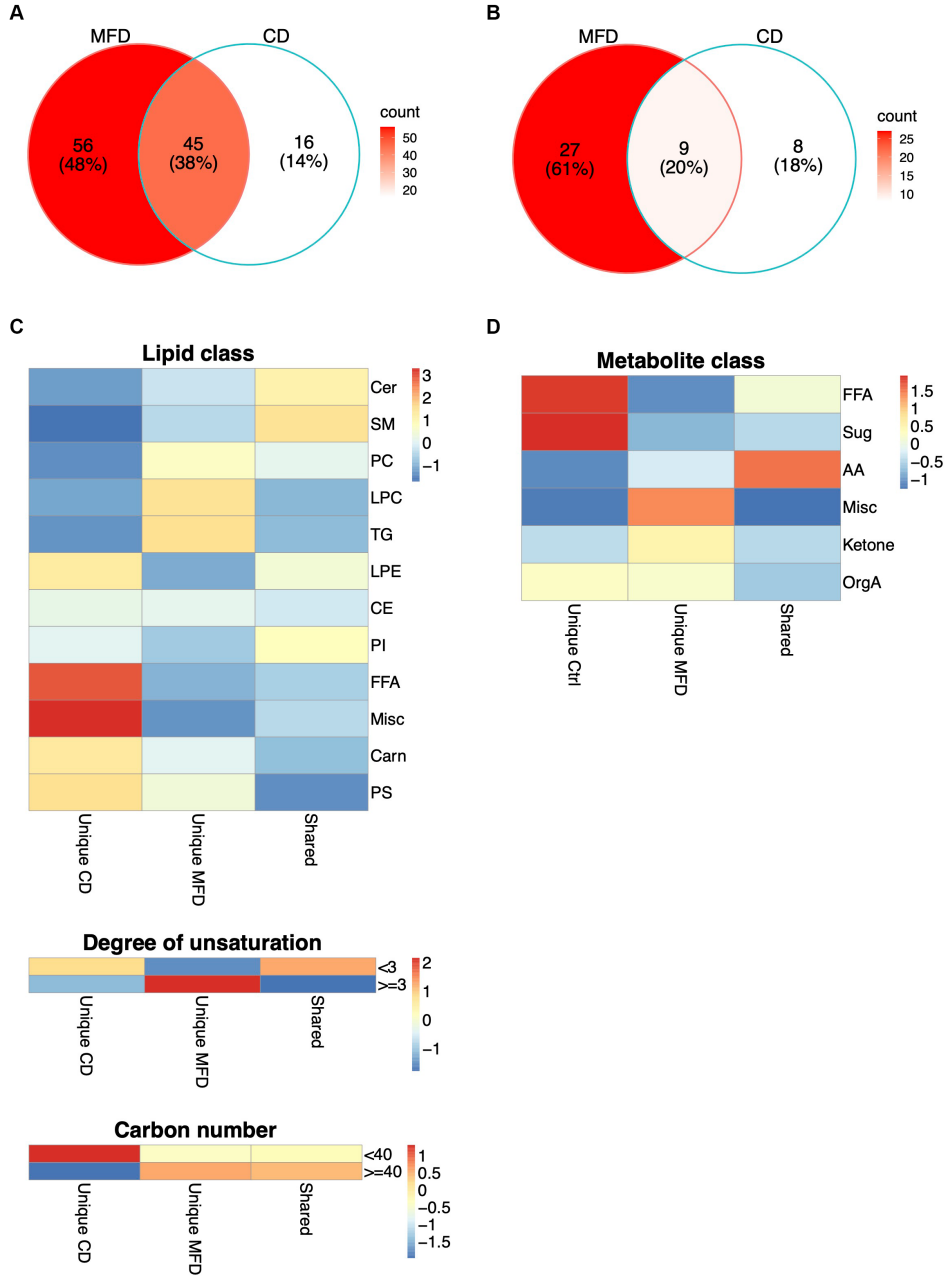
Shared and unique alterations in metabolism elicited by the MFD and the CD. **(A)** Alterations in lipid levels, and **(B)** and in metabolite levels unique to the MFD (red) and CD (white) or shared between diets (pink). **(C)** Distribution of shared and unique changes among lipid groups defined by lipid classes, degree of unsaturation (<3 or > =3) and acyl carbon number (<40 or > =40). Heatmaps show the difference between observed and expected levels, derived from a chi-squared test. **(D)** Distribution of shared and unique changes among metabolite groups, derived as explained in **(C)**.

Given the limited power to detect interactions in this relatively small sample, we also examined time-dependent alterations in metabolite and lipid levels in both diet groups independently. This revealed the MFD and CD to elicit altered levels of 101 and 61 lipids (Supplementary Table S4), and 36 and 17 low molecular weight metabolites (Supplementary Table S5), respectively. The lists of MFD- and CD-associated metabolites were then intersected and metabolites uniquely altered in any of the diets (unique) or altered in both diets (shared), identified. Out of the 117 lipids that showed altered levels in any of the diet groups, 56 were uniquely altered by the MFD, 16 uniquely altered by the CD and 45 shared between diets (Figure 2A; Supplementary Table S4). A total of 44 low molecular weight metabolites were altered by any of the diets, out of which 27 were uniquely altered by the MFD, 8 by the CD and 9 by both diets (Figure 2B; Supplementary Table S5). One out of the 54 shared (alanine), 8 of the 83 MFD unique (LPC20:2, TG54:4, PI36:1, proline, urea, glutamate, lactic acid and 2-hydroxybutyrate) and 7 of the CD unique (LPC18:2, SM40:2, PC36:2, chenodeoxycholic-, cholic-, and oleic acid) metabolites and lipids lost significance after adjustment for weight loss.

Clustering of lipids altered by the MFD revealed a general reduction in lipid and FA levels and a clear clustering of samples collected at baseline and at the follow-up visits at 4 and 8 weeks (Supplementary Figure S4). However, three clusters, mainly composed of lipids with a high carbon number and a high degree of unsaturation, showed elevated levels at follow up. Clustering was less clear for the CD, although 12 out of 20 of the baseline samples were clearly separated from the rest of the samples (Supplementary Figure S5). In contrast to the MFD, FAs were the only lipids that showed increased levels, whereas the majority of lipids that showed reduced levels had shorter acyls and a lower degree of unsaturation. Hence, these analyses suggested a systematic remodeling of the lipidome that depended on both acyl carbon number and degree of unsaturation. To examine this further, we grouped lipids by lipid class, carbon number (lower than or higher/equal to 40) and degree of unsaturation (lower than or higher/equal to 3) and examined the enrichment of these groups within diet unique and shared alterations in lipid levels. Lipid classes differed between groups (*p* = 0.00011), with pronounced alterations in FA and furan propionate levels in the CD and PC, LPC and TG levels in the MFD (Figure 2C; Supplementary Table S4). We also found a significant difference between diets with respect to degree of unsaturation (*p* = 0.00094) and carbon number (*p* = 0.040) dependent regulation of lipid levels. These analyses revealed that levels of shorter more saturated lipids mainly were increased in the CD and longer highly unsaturated lipids increased in the MFD (Figure 2C). A similar analysis in the metabolite data confirmed FA levels to be mainly increased by the CD. Additionally, these analyses revealed levels of a group of metabolites, including cholesterol and three glycerol-related metabolites, to be mainly increased in the MFD (Figure 2D; Supplementary Table S5).

**Figure 3:**
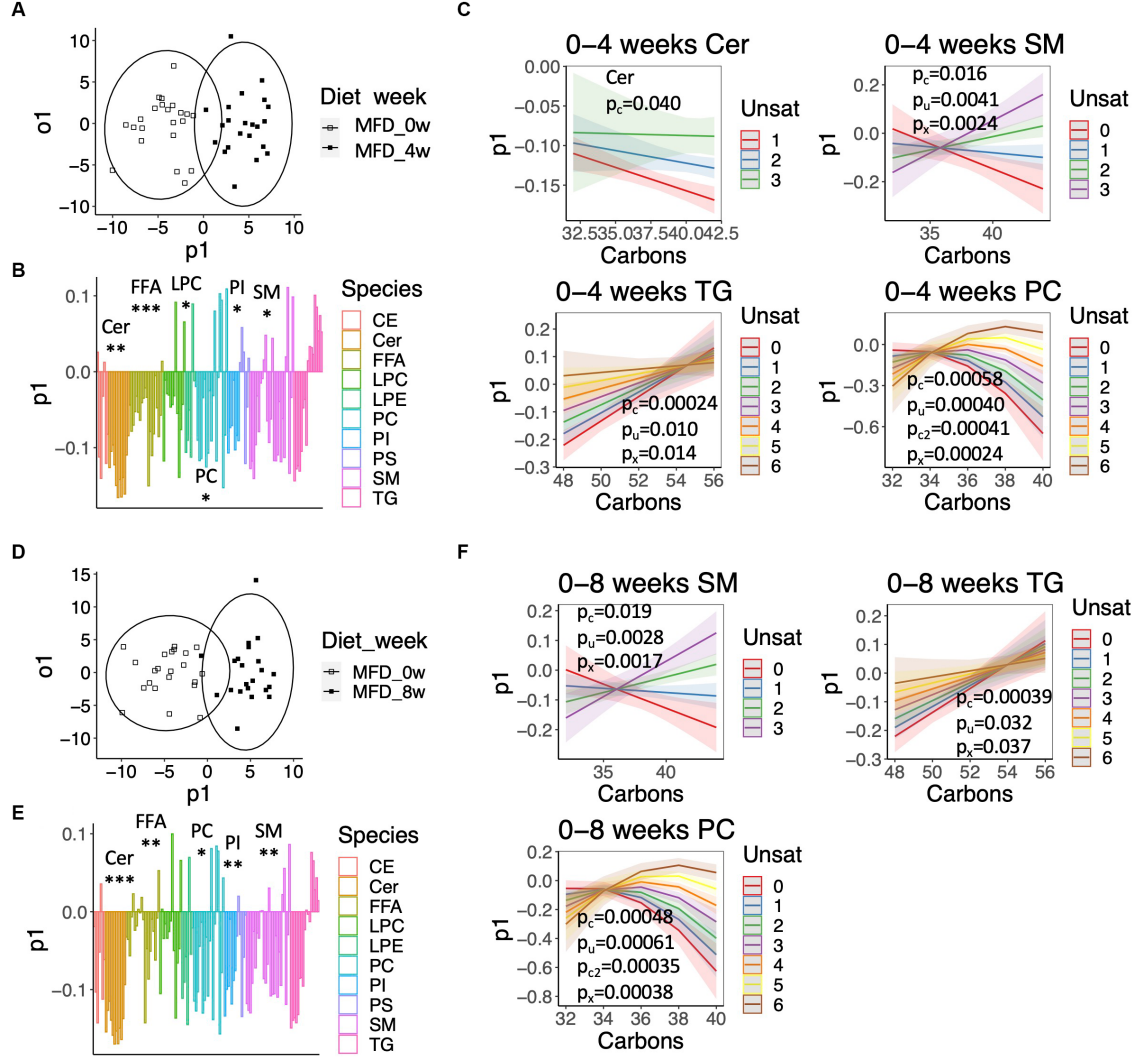
Systematic alterations in lipid and fatty acid levels elicited by the MFD. **(A)** OPLS-DA score-scatter plot showing a lipidome-based classification of samples collected at baseline and the 4-weeks study visit. **(B)** Loadings along the predictive component of the OPLS-DA model showed in **(A)** colored by lipid class. Significant class-wise alterations were assessed using chi-squared statistics: *q < 0.05, **q < 0.001, ***q < 0.0001. **(C)** Predicted values from regression models calculated on loadings for the indicated lipid classes. The shaded area shows the 95% confidence interval. Significant effects are given by: p_c_, p_u_, p_c2_, p_x_ for the number of carbons (Carbons), the degree of unsaturation (Unsat), Carbons^2^, and the interaction Carbons*Unsat. **(D)** Score-scatter plot for an OPLS-DA model calculated for the baseline and 8-weeks study visit. **(E)** Loadings along the predictive component of the OPLS-DA model showed in **(D)**, as described in **(B)**. **(F)** Predicted values calculated from loadings shown in **(E)**, as described in **(C)**.

Next, we examined systematic alterations in lipid levels separately in the CD and MFD groups using OPLS (Figure 3). The OPLS model calculated for the baseline and 4-weeks study visits resulted in a clear classification of samples for the MFD diet (R^2^_y_ = 0.80, Q^2^_y_ = 0.62, Figure 3A), which was largely driven by a systematic lowering of ceramide, FFA, LPC, PI, and SM species (Figure 3B); the model for the CD group was unreliable due to a significant degree of over fitting (R^2^_y_ = 0.69, Q^2^_y_ = 0.31). However, not all lipids within a class were similarly regulated. To examine this variation, we explored the loadings from the OPLS model as dependent variables and the carbon number and degree of unsaturation as independent variables in quadratic models including an interaction term. Changes in ceramide, SM, and TG species showed a linear dependence on the carbon number and degree of unsaturation, whereas PC species showed a non-linear dependence (Figure 3C). Overall, levels of long-chained ceramides, SMs and PCs with a low degree of unsaturation and relatively short-chained TGs with a low degree of unsaturation decreased the most. A similar result was observed for the MFD group when analyzing the baseline and 8-weeks study visit (R^2^_y_ = 0.80, Q^2^_y_ = 0.64) (Figures 3D–F); again, a model calculated for the CD was unreliable due to overfitting (R^2^_y_ = 0.75, Q^2^_y_ = 0.41).

Evaluation of structure-effect relationships among the low molecular weight metabolites measured by GC/MS is not as straightforward as for the lipids, given the heterogeneity of this group of compounds. However, notable changes in levels of metabolites related to lipid metabolism could be observed. For instance, levels of furan propionates, 3-carboxy-4-methyl-5-propyl-2-furanpropionate (CMPF), which is strongly associated with lipid metabolism, and its analog 3-carboxy-4-methyl-5-pentyl-2-furanpropionate (CMPeF), showed reduced levels in the CD group, with a slight, but transient, elevation in the MFD group at 4 weeks (Supplementary Figure S6A). Levels of glycerol-related metabolites were uniquely reduced in the MFD group (Supplementary Figure S6B).

### 3.4. Association between alterations in metabolite levels and clinical parameters

**Figure 4:**
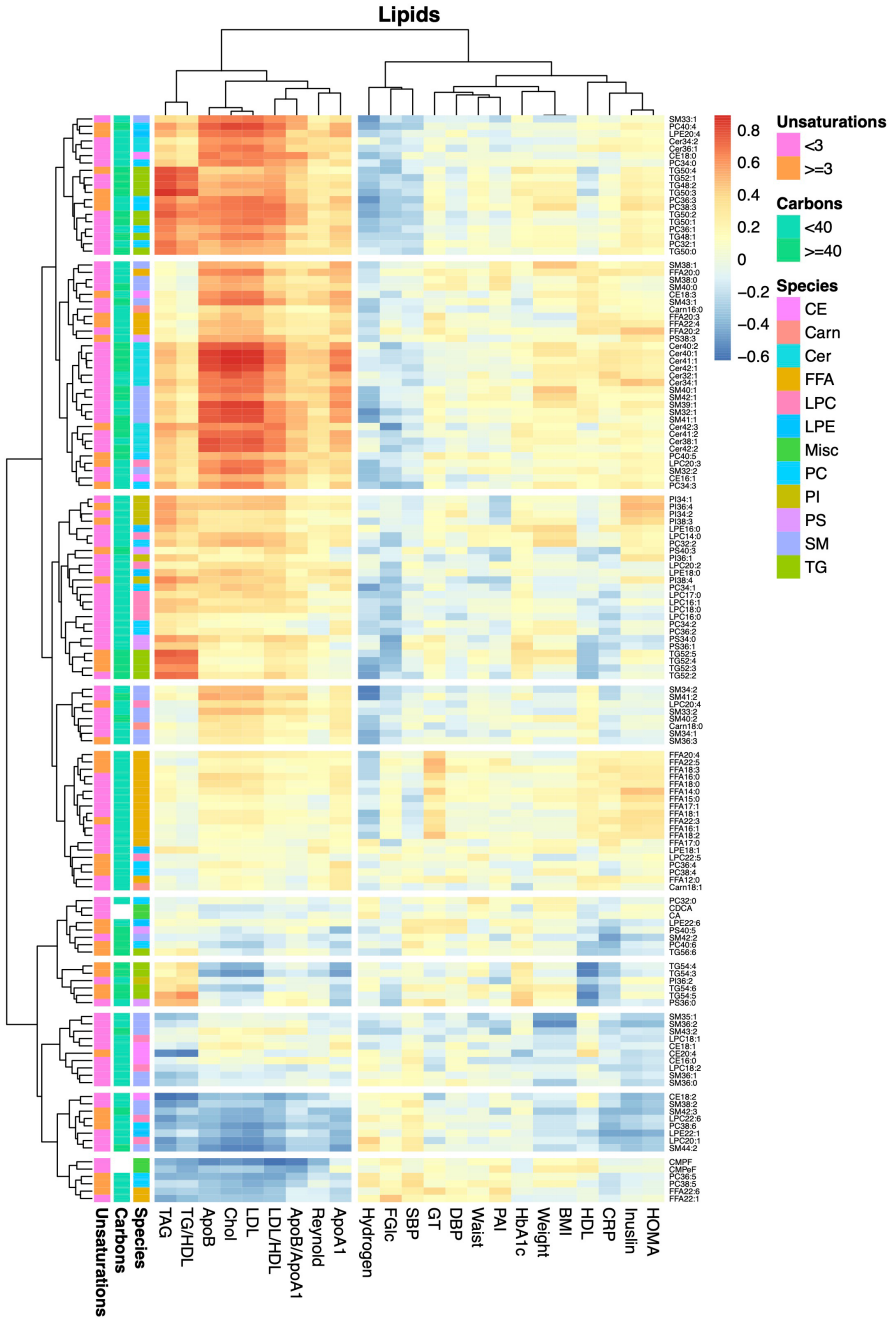
Heatmap showing correlations between alterations in lipids and anthropometric and blood chemistry data. The heatmap show pairwise Pearson correlations between alterations in lipid levels measured by the lipidomics protocol and changes in clinical chemical and anthropometric data between baseline and the 8-weeks study visit for both diet groups combined.

**Figure 5:**
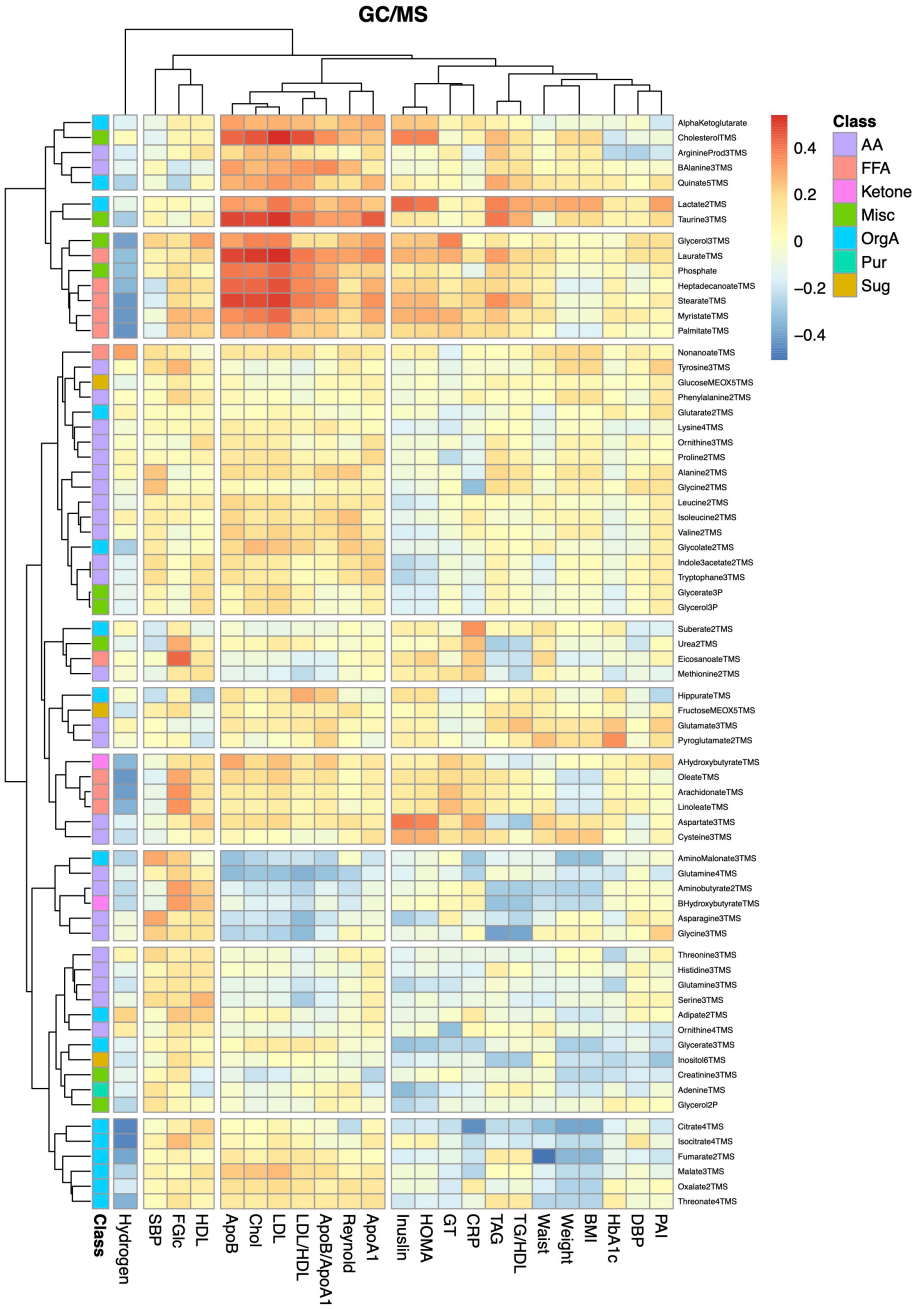
Heatmap showing correlations between alterations in metabolite and anthropometric and blood chemistry data. The heatmap show pairwise Pearson correlations between alterations in metabolites measured by GC/MS and changes in clinical chemical and anthropometric data between baseline and the 8-weeks study visit for both diet groups combined.

Finally, we examined associations between alterations in metabolite levels and clinical variables, including a number of established cardiovascular disease-risk markers, between the baseline visit and the 8-weeks follow-up visit for both diet groups combined. As expected, a large number of compounds in the lipidomics dataset (Figure 4; Supplementary Table S6) were associated with lipid and lipoprotein subclasses and their ratios. ApoA1, ApoB, cholesterol, TAG and LDL associated with 28, 71, 65, 61, and 64 lipids (*q* < 0.05), respectively. The 8 strongest associations between our lipidomic data and the TAG assay conducted in the clinic all included TAGs; in total 12 out of the 17 TAGs measured by our lipidomic platform associated with changes in total TAG levels. The strongest association with changes in total TAG levels was found for TAG50:3 (*r* = 0.87, *q* = 5.5e^−14^). Two ceramides, Cer41:1 (*r* = 0.78, *q* = 4.14e-13) and Cer40:1 (*r* = 0.77, *q* = 2.3e-12), showed impressively strong associations with LDL. However, several lipids, mainly involving those containing long and highly unsaturated acyls, such as PC36:5, PC38:6, LPC22:6, SM44:2, and TAG54:4, as well as furan propionates (CMPF and CMPeF), showed an inverse association with multiple lipid-related parameters. In line with the results from the analysis of the lipidomics dataset, analysis of the GC/MS dataset (Figure 5; Supplementary Table S7) revealed several associations between alterations in lipid-related metabolites, including lauric-, stearic-, and heptadecanoic-acid, and cholesterol, and changes in lipid parameters measured in the clinic, such as ApoB, cholesterol and LDL. Notably, changes in breath hydrogen excretion showed inverse associations with altered levels of myristic- (*r* = −0.42, *q* = 0.025), palmitic- (*r* = −0.43, *q* = 0.051), heptadecanoic- (*r* = −0.35, *q* = 0.25), stearic- (*r* = −0.42, *q* = 0.025) oleic- (*r* = −0.42, *q* = 0.0051), linoleic- (*r* = −0.36, *q* = 0.020), and arachidonic acid (*r* = −0.41, *q* = 0.0058), as well as aminobutyrate (*r* = −0.23, *q* = 0.025), beta-hydroxybutyrate (*r* = −0.23, *q* = 0.025) and glycerol (*r* = −0.040, *q* = 0.039).

Finally, we examined associations between altered lipid levels and changes in the Reynolds risk score, as a proxy for the risk of future cardiovascular disease. None of the lipids showed significant associations with the risk score after adjustment for multiple testing. Among the 29 lipids that showed nominally significant associations, only four showed an inverse association. These included CMPF (*r* = −0.39, *p* = 0.025), but also the long and/or highly unsaturated lipids PC38:6 (*r* = −0.33, *p* = 0.023), SM44:2 (*r* = −0.28, *p* = 0.023), and LPE22:1 (*r* = −0.21, *p* = 0.023). Among the metabolites detected by GC/MS, only lactate (*r* = 0.30, *p* = 0.015), beta-alanine (*r* = 0.26, *p* = 0.024) and cholesterol (*r* = 0.27, *p* = 0.039), showed nominally significant associations with the risk score.

## 4. Discussion

The concept of combining several foods with beneficial health effects into a MFD has been explored for more than a decade (5). Still, the mechanisms by which this diet promotes health remains partially enigmatic. In the first study on MFD in 2012, individuals in the treatment- and control-dietary periods were forced to be weight stable (5). Still, notable changes in metabolite levels could be observed between the two arms (7). Enforced weight maintenance is, however, not a condition observed in everyday life, especially not in situations when the diet is changed. Importantly, prevention of weight loss by alterations in calorie intake may potentially shield some diet-linked physiological effects. As a consequence, the present study was conducted without the requirement of weight stability. Interestingly, both the CD and MFD studied here resulted in significant weight loss over time, but there was no difference between groups (6). Hence, the present investigation is more in line with everyday living conditions, but still allows for investigation of weight loss-independent effects of an MFD.

Notably, alterations in lipid levels were more pronounced as compared to changes in levels of low molecular weight metabolites. Presumably, this observation may be explained by the higher short-term variability among small molecules, such as sugars and small organic acids, as compared to phospho-, sphingo-, and glycero-lipids (12). However, we cannot rule out that this observation relates to the selectivity of the GC/MS method. As compared to LC/MS-based methods, GC/MS only cover metabolites that become volatile upon derivatization, thereby excluding large parts of the low molecular weight metabolome from being detected.

Until recent years, the majority of metabolomics studies were focused on finding biomarkers centerd on single metabolites. Results from those studies have been somewhat disappointing, as very few biomarkers have been replicated. Major reasons for this shortcoming are the heterogeneity of applied analysis techniques, methods, and data analysis pipelines, as well as the high frequency of false positive discoveries burdening data produced by untargeted metabolomics (13). A plausible solution to this problem is to benefit from the co-variation structure that is observed in the metabolome as a consequence of metabolism being a highly integrated network (14). Systematic patterns, involving multiple covarying metabolites, are more likely to reflect a true metabolic effect as compared to alterations in levels of a single metabolite.

Hence, to benefit from the covariation structure in the data, we used OPLS to explore treatment-effect patterns. An advantage of OPLS over univariate methods and the more commonplace PLS method is its capability to eliminate biases in the analyses caused by the existence of systematic outcome independent variation in the data (15). Robust models, classifying samples acquired at baseline and follow-up, could be constructed for the MFD but not for the CD, emphasizing a more pronounced and systematic metabolic remodulation in the former despite of a similar weight-loss. Moreover, these analyses revealed that the MFD-elicited alterations in the lipidome depended on the lipid class, acyl carbon number and degree of unsaturation. More specifically, we found that levels of lipids with shorter acyl-chain lengths and a lower degree of unsaturation were decreased by the MFD, whereas levels of those with longer acyl-chain lengths and a higher degree of unsaturation increased. These findings are in line with a previous study on MFD where lipids having acyls with a degree of unsaturation less than 3 were found to decrease, whereas those containing acyls with 5 or more unsaturations increased (7). Interestingly, this pattern has also been observed after bariatric surgery (8), and linked with decreased insulin resistance and a reduced future risk of T2D (16). Hence, metabolite patterns derived from different studies can be compared, despite of different analysis methods being used and different lipid species being detected. Whether the observed lipid remodulation is caused by the higher content of fish and rapeseed oil in the MFD as compared to the CD (5), or being a secondary effect caused by alterations in desaturase and elongase activity remains to be established. *In vivo* elongation and desaturation of fatty acids occurs in parallel with *de novo* lipogenesis (17), allowing cells to locally adjust availability of specific lipids to balance the lipid pool (18). Hence, the body encompasses a dynamic system for modulation of the lipid composition and quite dramatic alterations in dietary fat composition are required to produce even very small alterations the lipidome (19). Previous studies have found insulin resistance to associate with a reduced activity of delta-9 and -6 desaturases (20). Also, fatty acid elongation has been implicated in metabolic disease, as exemplified by variation in fatty acid elongase 2 (Elovl2), which has been linked to reduced expression of the enzyme, diminished production of unsaturated fatty acids and a higher BMI (21). On the contrary, Elovl6, which catalyzes the transformation of palmitate to stearate, has been shown to contribute to insulin resistance (22). Clearly, elucidation of the exact mechanism underlying the observed alterations in lipid profiles requires establishment of robust biomarker patterns, rather than single biomarkers, in addition to a thorough understanding of the selectivity of plausible enzymatic mediators. This notwithstanding, the inverse association between lipids containing long and polyunsaturated acyls and the Reynolds risk score confirms a beneficial effect of the observed lipid remodulation.

After having established a systematic effect of the MFD on the lipid pattern, the fundamental question remains unresolved: What is the mechanism underlying potential health-promoting effects of increased fatty acid acyl length and degree of unsaturation? The previously proposed “membrane theory of diabetes” may provide some clues (19). This theory proposes that increased levels of saturated fatty acids, via either the diet or *de novo* synthesis from carbohydrates, leads to the formation of rigid cellular membranes and a reduction of membrane fluidity. In turn, such membranes adversely impact on multiple physiological functions, including insulin signaling, glucose uptake and blood circulation. In support of this is the abnormal rigidity of many different cell types that is observed in individuals with diabetes (19), which has been associated with a reduced membrane fluidity (23), and, in turn, excess saturated fatty acids (24). Contrary to the effect of the degree of unsaturation on membrane fluidity, fatty acid elongation is expected to increase the rigidity of the membrane (25). However, the effect of acyl chain-length also depend on the degree of unsaturation (25), which can be understood from the consecutive action of elongases and desaturases (18). Our data support this by identifying strong interactions between fatty acyl chain lengths and degree of unsaturation.

It is possible that the observed metabolic regulation is just reflecting a differential impact of the MFD and the CD on the catabolic state. However, against this hypothesis stands the observation of FFA levels being uniquely increased by the CD, despite weight loss being identical between the two diet groups. Levels of FFAs are expected to increase acutely in response to weight loss and starvation (26), but then reach sub-baseline levels with time when weight loss stabilizes (27). In line with this, levels of acylcarnitines decreased uniquely after the MFD, which is opposite to what is observed after prolonged starvation, in particular for the long-chained acylcarnitines measured in the present study (8, 26). This observation is also in agreement with a previous study on the MFD performed under the condition of weight-maintenance (7). Hence, our data do not support a difference in catabolism between the two diet groups.

In addition to the systematic alterations in lipid levels, we also found notable alterations in levels of several other metabolites. For instance, levels of CMPF increased during the first 4-weeks of the MFD, whereas levels of both CMPF and CMPeF decreased in individuals consuming the CD. Interestingly, CMPF showed a nominal inverse association with the Reynolds risk score. Elevated plasma levels of CMPF and CMPeF have been associated with fish-intake (28) and consumption of a Mediterranean diet (29), respectively. The question of whether alterations in levels of these molecules merely reflect the composition of the diet or if they also mediate some of the beneficial health effects associated with these diets remains to be resolved.

We found a large number of strong associations between changes in lipid levels and alterations in traditional lipid variables, such as LDL and apolipoprotein levels. The strongest association was observed for changes in ceramide levels and altered levels of LDL. Ceramides have been shown to parallel increased levels of LDL in diabetes (30) and to predict heart disease with a similar power as LDL (31). Moreover, weight loss-elicited alterations in multiple ceramide species have been found to associate with changes in LDL levels (32), although the correlations were much weaker as compared to those found in the present investigation.

Notably, a number of low-molecular weight metabolites, mainly fatty acids and glycerol, showed negative associations with fasting breath hydrogen levels. Hydrogen is produced when anaerobic bacteria produce short chain fatty acids from non-digested carbohydrates in the large intestine, a process that has been associated with multiple positive effects on health (33). Hence, the increased hydrogen excretion observed with the MFD (6) is likely due to the higher dietary fiber content of the MFD, as compared to the CD. Short-chain fatty acids impact on many branches of lipid metabolism, one being activation of AMP-activated protein kinase (AMPK), which via downstream processes leads to upregulation of lipolysis, beta-oxidation and ketone body metabolism, thereby resulting in a lowering of circulating glycerol and fatty acid levels (34). We also found an inverse association between breath hydrogen and aminobutyric acid. Interestingly, *Bacteroides* have been shown to produce aminobutyric acid (35), which is in line with the increased *Prevotella/Bacteroides* ratio observed in individuals consuming MFD (4). Some care has to be taken when interpreting the breath hydrogen test as about 15–30% of the population harbor *Methanobrevibacter smithii*, which convert hydrogen gas to methane, and as some individuals do not produce hydrogen gas at all (36).

Some limitations of the study merits mentioning. Firstly, the time-span for the study is limited to 8 weeks. Even though 8 weeks is much longer than previous studies (5, 7), it is still far from the time-scale required to accurately measure weight changes, which in the general population may occur at a rate of 500 g per year (37). Hence, it is still possible that the impact of the diets on long-term weight development differ. However, diet-elicited effects on the metabolic state would be expected to be reflected in the plasma metabolome and the absence of a catabolic state-associated metabolite profile suggests that this is not the case. Secondly, the study is descriptive in its nature. Identification of causalities and characterization of effects elicited by all nutrients and functional components in the MFD, together with all possible synergies, is a great commitment. Thirdly, although the study involved both men and women, the relatively small sample size and the predominance of women in both groups, precludes analysis of sex-dependent metabolic effects of the MFD. Finally, it is well known that the genetic background influences the response to diets. This is something that should be considered in future studies, as the present study lacks such data and is underpowered for such analyses.

## 5. Conclusion

In conclusion, focusing on metabolite and lipid patterns, rather than individual lipids and metabolites, allowed us to compare results from the present investigation with results from previous studies in which other metabolites and lipids were detected, thereby supporting a beneficial health effect of the MFD. Whether the health-promoting lipid signature, which is also observed in other weight loss interventions (8, 16), is targetable via modulation desaturase and elongase activity remains to be elucidated.

## Data availability statement

The raw data supporting the conclusions of this article will be made available by the authors, without undue reservation.

## Ethics statement

The studies involving humans were approved by the Regional Ethical Review Board, Lund, Sweden. The studies were conducted in accordance with the local legislation and institutional requirements. The human samples used in this study were acquired from a previous study for which ethical approval was obtained (Dnr 2013/584). Written informed consent for participation was obtained from the participants in accordance with the national legislation and institutional requirements.

## Author contributions

CA and MA-M: investigation. MO and OR: investigation and formal analysis. IB and JT: conceptualization, methodology, and writing–reviewing and editing. PS: conceptualization, methodology, formal analysis, investigation, writing–original draft, writing–reviewing and editing, and visualization. All authors contributed to the article and approved the submitted version.

## Funding

This study was supported by the Lund University Antidiabetic Food Center (AFC), a VINNOVA VINN Excellence Center, the Hjelt Foundation, the Novo Nordisk Foundation, the Albert Påhlsson Foundation, the P. Håkansson’s Foundation, and the Swedish Diabetes Foundation. The funders had no role in the study design, data collection or analysis, decision to publish, or preparation of the manuscript.

## Conflict of interest

The authors declare that the research was conducted in the absence of any commercial or financial relationships that could be construed as a potential conflict of interest.

## Publisher’s note

All claims expressed in this article are solely those of the authors and do not necessarily represent those of their affiliated organizations, or those of the publisher, the editors and the reviewers. Any product that may be evaluated in this article, or claim that may be made by its manufacturer, is not guaranteed or endorsed by the publisher.
