# Supplementary material for: A randomized trial involving a multifunctional diet reveals systematic lipid remodeling and improvements in cardiometabolic risk factors in middle aged to aged adults
Source: Front Nutr. 2023 Sep 14;10:1236153. doi: 10.3389/fnut.2023.1236153 (PMC10538628; doi:10.3389/fnut.2023.1236153)
Supplement: Supplementary file 1 [file Data_Sheet_1.docx]

Supplementary Material

A randomised trial involving a multifunctional diet reveals systematic lipid remodelling and improvements in cardiometabolic risk factors

Claudia Balderas Arroyo^1^, Maider Greño Ocariz^1^, Oksana Rogova^1^, Mahmoud Al-Majdoub^2^, Inger Björck^3^, Juscelino Tovar^4^ , and Peter Spégel^1, *^

*** Correspondence:** Peter Spégel: [peter.spegel@chem.lu.se](mailto:peter.spegel@chem.lu.se)

**Supplement Pages**

Supplemental Table S1 2

Supplemental Table S2 3-6

Supplemental Table S3 7-8

Supplemental Table S4 9-13

Supplemental Table S5 14-15

Supplemental Table S6 16-55

Supplemental Table S7 56-71

Supplemental Figure S1 72

Supplemental Figure S2 73-76

Supplemental Figure S3 77

Supplemental Figure S4 78

Supplemental Figure S5 79

Supplemental Figure S6 80

Supplemental Figure S7 81

**Table S1**. Average contribution of main food categories to CD and MFD (g/day).

|  | **CD** | | **MFD** | |
| --- | --- | --- | --- | --- |
|  | **Women** | **Men** | **Women** | **Men** |
| Fish | 32 | 32 | 78 | 85 |
| Whole grains | 14 | 14 | 87 | 87 |
| Fruits and vegetables | 731 | 765 | 850 | 890 |
| Dairy | 325 | 370 | 12 | 15 |
| Red and processed meat | 97 | 112 | 56 | 46 |

**Table S2**. Results from the LMM analyses of the lipid data. Page 1(4)

**Table S2**. Results from the LMM analyses of the lipid data. Page 2(4)

**Table S2**. Results from the LMM analyses of the lipid data. Page 3(4)

**Table S2**. Results from the LMM analyses of the lipid data. Page 4(4)

**Table S3.** Results from the LMM analyses of the metabolite data. Page 1(2).

**Table S3.** Results from the LMM analyses of the metabolite data. Page 2(2).

**Table S4.** Results from independent analyses of lipids (lipidomics) in the MFD and CD group using LMM. Lipids are indicated as being either uniquely altered in the MFD (MFD, in the CD (CD), altered in both diets (Shared) or unaltered in both diets (ns). Page 1(5).

**Table S4.** Results from independent analyses of lipids (lipidomics) in the MFD and CD group using LMM. Lipids are indicated as being either uniquely altered in the MFD (MFD, in the CD (CD), altered in both diets (Shared) or unaltered in both diets (ns). Page 2(5).

**Table S4.** Results from independent analyses of lipids (lipidomics) in the MFD and CD group using LMM. Lipids are indicated as being either uniquely altered in the MFD (MFD, in the CD (CD), altered in both diets (Shared) or unaltered in both diets (ns). Page 3(5).

**Table S4.** Results from independent analyses of lipids (lipidomics) in the MFD and CD group using LMM. Lipids are indicated as being either uniquely altered in the MFD (MFD, in the CD (CD), altered in both diets (Shared) or unaltered in both diets (ns). Page 4(5).

**Table S4.** Results from independent analyses of lipids (lipidomics) in the MFD and CD group using LMM. Lipids are indicated as being either uniquely altered in the MFD (MFD, in the CD (CD), altered in both diets (Shared) or unaltered in both diets (ns). Page 5(5).

**Table S5**. Results from independent analyses of metabolites (GC/MS) in the MFD and CD group using LMM. Metabolites are indicated as being either uniquely altered in the MFD (MFD, in the CD (CD), altered in both diets (Shared) or unaltered in both diets (ns). Page 1(2).

**Table S5**. Results from independent analyses of metabolites (GC/MS) in the MFD and CD group using LMM. Metabolites are indicated as being either uniquely altered in the MFD (MFD, in the CD (CD), altered in both diets (Shared) or unaltered in both diets (ns). Page 2(2).

**Table S6**. Results from pairwise Spearman correlations between alterations in lipidomics data and changes in clinical chemical variables. Page 1(40).

**Table S6**. Results from pairwise Spearman correlations between alterations in lipidomics data and changes in clinical chemical variables. Page 2(40).

**Table S6**. Results from pairwise Spearman correlations between alterations in lipidomics data and changes in clinical chemical variables. Page 3(40).

**Table S6**. Results from pairwise Spearman correlations between alterations in lipidomics data and changes in clinical chemical variables. Page 4(40).

**Table S6**. Results from pairwise Spearman correlations between alterations in lipidomics data and changes in clinical chemical variables. Page 5(40).

**Table S6**. Results from pairwise Spearman correlations between alterations in lipidomics data and changes in clinical chemical variables. Page 6(40).

**Table S6**. Results from pairwise Spearman correlations between alterations in lipidomics data and changes in clinical chemical variables. Page 7(40).

**Table S6**. Results from pairwise Spearman correlations between alterations in lipidomics data and changes in clinical chemical variables. Page 8(40).

**Table S6**. Results from pairwise Spearman correlations between alterations in lipidomics data and changes in clinical chemical variables. Page 9(40).

**Table S6**. Results from pairwise Spearman correlations between alterations in lipidomics data and changes in clinical chemical variables. Page 10(40).

**Table S6**. Results from pairwise Spearman correlations between alterations in lipidomics data and changes in clinical chemical variables. Page 11(40).

**Table S6**. Results from pairwise Spearman correlations between alterations in lipidomics data and changes in clinical chemical variables. Page 12(40).

**Table S6**. Results from pairwise Spearman correlations between alterations in lipidomics data and changes in clinical chemical variables. Page 13(40).

**Table S6**. Results from pairwise Spearman correlations between alterations in lipidomics data and changes in clinical chemical variables. Page 14(40).

**Table S6**. Results from pairwise Spearman correlations between alterations in lipidomics data and changes in clinical chemical variables. Page 15(40).

**Table S6**. Results from pairwise Spearman correlations between alterations in lipidomics data and changes in clinical chemical variables. Page 16(40).

**Table S6**. Results from pairwise Spearman correlations between alterations in lipidomics data and changes in clinical chemical variables. Page 17(40).

**Table S6**. Results from pairwise Spearman correlations between alterations in lipidomics data and changes in clinical chemical variables. Page 18(40).

**Table S6**. Results from pairwise Spearman correlations between alterations in lipidomics data and changes in clinical chemical variables. Page 19(40).

**Table S6**. Results from pairwise Spearman correlations between alterations in lipidomics data and changes in clinical chemical variables. Page 20(40).

**Table S6**. Results from pairwise Spearman correlations between alterations in lipidomics data and changes in clinical chemical variables. Page 21(40).

**Table S6**. Results from pairwise Spearman correlations between alterations in lipidomics data and changes in clinical chemical variables. Page 22(40).

**Table S6**. Results from pairwise Spearman correlations between alterations in lipidomics data and changes in clinical chemical variables. Page 23(40).

**Table S6**. Results from pairwise Spearman correlations between alterations in lipidomics data and changes in clinical chemical variables. Page 24(40).

**Table S6**. Results from pairwise Spearman correlations between alterations in lipidomics data and changes in clinical chemical variables. Page 25(40).

**Table S6**. Results from pairwise Spearman correlations between alterations in lipidomics data and changes in clinical chemical variables. Page 26(40).

**Table S6**. Results from pairwise Spearman correlations between alterations in lipidomics data and changes in clinical chemical variables. Page 27(40).

**Table S6**. Results from pairwise Spearman correlations between alterations in lipidomics data and changes in clinical chemical variables. Page 28(40).

**Table S6**. Results from pairwise Spearman correlations between alterations in lipidomics data and changes in clinical chemical variables. Page 29(40).

**Table S6**. Results from pairwise Spearman correlations between alterations in lipidomics data and changes in clinical chemical variables. Page 30(40).

**Table S6**. Results from pairwise Spearman correlations between alterations in lipidomics data and changes in clinical chemical variables. Page 31(40).

**Table S6**. Results from pairwise Spearman correlations between alterations in lipidomics data and changes in clinical chemical variables. Page 32(40).

**Table S6**. Results from pairwise Spearman correlations between alterations in lipidomics data and changes in clinical chemical variables. Page 33(40).

**Table S6**. Results from pairwise Spearman correlations between alterations in lipidomics data and changes in clinical chemical variables. Page 34(40).

**Table S6**. Results from pairwise Spearman correlations between alterations in lipidomics data and changes in clinical chemical variables. Page 35(40).

**Table S6**. Results from pairwise Spearman correlations between alterations in lipidomics data and changes in clinical chemical variables. Page 36(40).

**Table S6**. Results from pairwise Spearman correlations between alterations in lipidomics data and changes in clinical chemical variables. Page 37(40).

**Table S6**. Results from pairwise Spearman correlations between alterations in lipidomics data and changes in clinical chemical variables. Page 38(40).

**Table S6**. Results from pairwise Spearman correlations between alterations in lipidomics data and changes in clinical chemical variables. Page 39(40).

**Table S6**. Results from pairwise Spearman correlations between alterations in lipidomics data and changes in clinical chemical variables. Page 40(40).

**Table S7.** Results from pairwise Spearman correlations between alterations in metabolomics (GC/MS) data and changes in clinical chemical variables. Page 1(16).

**Table S7.** Results from pairwise Spearman correlations between alterations in metabolomics (GC/MS) data and changes in clinical chemical variables. Page 2(16).

**Table S7.** Results from pairwise Spearman correlations between alterations in metabolomics (GC/MS) data and changes in clinical chemical variables. Page 3(16).

**Table S7.** Results from pairwise Spearman correlations between alterations in metabolomics (GC/MS) data and changes in clinical chemical variables. Page 4(16).

**Table S7.** Results from pairwise Spearman correlations between alterations in metabolomics (GC/MS) data and changes in clinical chemical variables. Page 5(16).

**Table S7.** Results from pairwise Spearman correlations between alterations in metabolomics (GC/MS) data and changes in clinical chemical variables. Page 6(16).

**Table S7.** Results from pairwise Spearman correlations between alterations in metabolomics (GC/MS) data and changes in clinical chemical variables. Page 7(16).

**Table S7.** Results from pairwise Spearman correlations between alterations in metabolomics (GC/MS) data and changes in clinical chemical variables. Page 8(16).

**Table S7.** Results from pairwise Spearman correlations between alterations in metabolomics (GC/MS) data and changes in clinical chemical variables. Page 9(16).

**Table S7.** Results from pairwise Spearman correlations between alterations in metabolomics (GC/MS) data and changes in clinical chemical variables. Page 10(16).

**Table S7.** Results from pairwise Spearman correlations between alterations in metabolomics (GC/MS) data and changes in clinical chemical variables. Page 11(16).

**Table S7.** Results from pairwise Spearman correlations between alterations in metabolomics (GC/MS) data and changes in clinical chemical variables. Page 12(16).

**Table S7.** Results from pairwise Spearman correlations between alterations in metabolomics (GC/MS) data and changes in clinical chemical variables. Page 13(16).

**Table S7.** Results from pairwise Spearman correlations between alterations in metabolomics (GC/MS) data and changes in clinical chemical variables. Page 14(16).

**Table S7.** Results from pairwise Spearman correlations between alterations in metabolomics (GC/MS) data and changes in clinical chemical variables. Page 15(16).

**Table S7.** Results from pairwise Spearman correlations between alterations in metabolomics (GC/MS) data and changes in clinical chemical variables. Page 16(16).

**Supplementary Figure 1.** Consort flow diagram.

**Supplementary Figure 2.** Lipids showing a significant time*diet interactions in the LMM analyses. Data are centred and scaled to unit variance. The paired Student’s t-test was used for post hoc significance testing. *p<0.05, **p<0.01, ***p<0.001, ****p<0.0001, ns, not significant. Numbers following diet type indicate time-point: 1, baseline; 2, 4-weeks; 3, 8-weeks. Page 1(4).

**Supplementary Figure 2.** Lipids showing a significant time*diet interactions in the LMM analyses. Data are centred and scaled to unit variance. The paired Student’s t-test was used for post hoc significance testing. *p<0.05, **p<0.01, ***p<0.001, ****p<0.0001, ns, not significant. Numbers following diet type indicate time-point: 1, baseline; 2, 4-weeks; 3, 8-weeks. Page 2(4).

**Supplementary Figure 2.** Lipids showing a significant time*diet interactions in the LMM analyses. Data are centred and scaled to unit variance. The paired Student’s t-test was used for post hoc significance testing. *p<0.05, **p<0.01, ***p<0.001, ****p<0.0001, ns, not significant. Numbers following diet type indicate time-point: 1, baseline; 2, 4-weeks; 3, 8-weeks. Page 3(4).

**Supplementary Figure 2.** Lipids showing a significant time*diet interactions in the LMM analyses. Data are centred and scaled to unit variance. The paired Student’s t-test was used for post hoc significance testing. *p<0.05, **p<0.01, ***p<0.001, ****p<0.0001, ns, not significant. Numbers following diet type indicate time-point: 1, baseline; 2, 4-weeks; 3, 8-weeks. Page 4(4).

**Supplementary Figure 3** Metabolites showing a significant time*diet interactions in the LMM analyses. Data are centred and scaled to unit variance. The paired Student’s t-test was used for post hoc significance testing. *p<0.05, **p<0.01, ***p<0.001, ****p<0.0001, ns, not significant. Numbers following diet type indicate time-point: 1, baseline; 2, 4-weeks; 3, 8-weeks.

**Supplementary Figure 4.**  Heatmap showing clustering of lipids that are significantly altered in the MFD group. Differences between individuals were removed prior to clustering using a linear model.

**Supplementary Figure 5.** Heatmap showing clustering of lipids that are significantly altered in the CD group. Differences between individuals were removed prior to clustering using a linear model.

**Supplementary Figure 6.** Changes in levels of lipids between baseline and 8 weeks follow up and its dependence on carbon number, degree of unsaturation and diet type. Each row corresponds to a lipid class. The left column shows alterations in lipid levels in relation to carbon number and the right column alterations in relation to the degree of unsaturation. Blue solid line illustrates the CD and the red line the MFD. The shaded area shows the 95% confidence intervals. P-values are provided for the effect of diet (p_d_), carbon number (p_c_), degree of unsaturation (p_u_), and the interaction (p_x_) between diet*carbon and diet*degree of unsaturation, for the left and right column, respectively. ns, not significant.

**Supplementary Figure 7.** Alteration in furan propionates and glycerol-related metabolites in the MFD and CD. **(a)** Furanpropionates, 3-carboxy-4-methyl-5-propyl-2-furanpropionate (CMPF) and 3-carboxy-4-methyl-5-pentyl-2-furanpropionate (CMPeF) are mainly altered in the CD. **(b)** Glycerol, glycerol 3-phosphate (Glycerol3P) and 3-phosphoglycerate (Glycerate3P) are only altered in the MFD. Graphs show centred and unit variance scaled relative levels of metabolites. Significance tested by LMM and the paired Student’s t-test *post hoc*. *p<0.05, **p<0.01, ***p<0.001, ****p<0.0001, ns, not significant.
